# Detection of a Tumor Suppressor Gene Variant Predisposing to Colorectal Cancer in an 18th Century Hungarian Mummy

**DOI:** 10.1371/journal.pone.0147217

**Published:** 2016-02-10

**Authors:** Michal Feldman, Israel Hershkovitz, Ella H. Sklan, Gila Kahila Bar-Gal, Ildikó Pap, Ildikó Szikossy, Rina Rosin-Arbesfeld

**Affiliations:** 1 Department of Anatomy and Anthropology, Sackler Faculty of Medicine, Tel-Aviv University, Tel-Aviv, Israel; 2 Department of Clinical Microbiology and Immunology, Sackler Faculty of Medicine, Tel-Aviv University, Tel-Aviv, Israel; 3 Koret School of Veterinary Medicine, The Robert H. Smith faculty of Agriculture, Food and Environment, The Hebrew University of Jerusalem, Rehovot, Israel; 4 Department of Anthropology, Hungarian Natural History Museum, Budapest, Hungary; University of Florence, ITALY

## Abstract

Mutations of the Adenomatous polyposis coli (*APC*) gene are common and strongly associated with the development of colorectal adenomas and carcinomas. While extensively studied in modern populations, reports on visceral tumors in ancient populations are scarce. To the best of our knowledge, genetic characterization of mutations associated with colorectal cancer in ancient specimens has not yet been described. In this study we have sequenced hotspots for mutations in the APC gene isolated from 18^th^ century naturally preserved human Hungarian mummies. While wild type APC sequences were found in two mummies, we discovered the E1317Q missense mutation, known to be a colorectal cancer predisposing mutation, in a large intestine tissue of an 18^th^ century mummy. Our data suggests that this genetic predisposition to cancer already existed in the pre-industrialization era. This study calls for similar investigations of ancient specimens from different periods and geographical locations to be conducted and shared for the purpose of obtaining a larger scale analysis that will shed light on past cancer epidemiology and on cancer evolution.

## Introduction

Colorectal cancer is a major cause of morbidity and mortality in western countries [1,2]. Improvements in early detection and treatment have resulted in decline of mortality rates while incidence rates have been increasing [3]. Typically, the precursors for colorectal cancer are adenomatous polyps, which are benign neoplastic clumps of cells [4]. Most sporadic adenomatous polyps as well as most colorectal cancers contain typical genetic alterations [5]. *Adenomatous polyposis coli (APC)* is an important tumor suppressor gene that is located on the human chromosome 5q21. Mutations in APC are strongly associated with the development of colorectal adenomas and carcinomas [6]. About 50% of the population will develop colorectal polyps initiated by such mutations during a normal life span [7]. Somatic mutations of the APC gene were detected not only in patients with colorectal carcinoma, but also in patients with pancreatic cancer [8], gastric cancer [9], oral squamous cell carcinoma [10], hepatoblastoma, breast cancer, brain tumor and desmoid tumor [11]. About 80% of somatic mutations of the APC gene occur in specific “hot spot” and are clustered within a region from codon 764 to codon 1596 called the Mutation Cluster Region (MCR). More than 95% are chain-terminating mutations that would result in the expression of truncated protein. Inactivation of both alleles of APC is required for development of most tumors in the colon and rectum [12].

Cancer has early documentations. Egyptian medical papyri dating as far back as 1500 BCE have been found to describe tumors. Herodotus and Hippocrates both mention cancer [13–15]. Most paleopathological reports on tumors in past populations are based on skeletal tissue which is more abundant in archaeological sites. However, some tumors in soft tissue have been reported [16–26]. While there are many theories regarding the prevalence of cancer in our days, which associate cancer with life style, diet, physical inactivity and reproductive patterns, more information from different time points in history is needed to better understand the role of these factors in historical populations.

Natural mummification enables preservation of soft tissue. Samples from mummified tissues can provide invaluable information from anthropological, historical and medical points of view. They can teach us important lessons regarding the evolution of diseases that might be of value for predicting future evolutionary changes. In 1994 and 1995 over 265 mummies were excavated from sealed crypts in the Dominican church in Vác, Hungary. The crypts were used continuously for burials of several middle-class families and clerics, from 1731–1838. The temperature in the crypts ranged between eight to eleven degrees Celsius, the crypts were poorly but continually ventilated and the remains were protected against humidity by pine shavings that filled many of the coffins. These were ideal conditions for natural preservation causing approximately 70% of the bodies to be totally or partially mummified. The preservation level of the mummified tissue samples and abundant contemporaneous archival information about the individuals of the Hungarian mummy collection motivated a morphological and genetic study of the human remains [27]. Previous studies found genetic evidence of *Mycobacterium tuberculosis* (*M*. *tuberculosis*) presence in these mummies [28–34], indicating that this cohort can be used for genetic studies. In addition, this cohort comprises of individuals that form a wide age distribution. Thus, it is compatible with the study of cancer associated mutations, as the risk for such mutations increases with age [3]. Here we used the Vác mummies to assess the existence of genetic predisposition to colorectal cancer in the pre-industrialization era by sequencing of “hot spots” in the APC gene. Three such sequences were amplified and sequenced from 3 different mummies. The APC variant E1317Q, known to predispose to colorectal cancer was detected in a colon sample of one mummy. While only a few APC sequences were obtained the presence of the E1317Q variant in the DNA of an 18^th^ century individual suggests that genetic predisposition to cancer already existed in the pre-industrialization era. This study however calls for a larger scale analysis for epidemiological comparison purposes.

## Materials and Methods

### Samples and precautions against contamination

The 18th century „Vác Mummy Collection” is housed and curated in the Department of Anthropology of the Hungarian Natural History Museum, in Budapest, Hungary. The collection contains 265 naturally mummified, partially mummified and skeletal specimens (registered under the Inventory numbers: 2009.19.1–2009.19.264). A total of 51 samples were obtained from 20 Vác mummies (Table 1). The samples were collected in the Anthropology Department of the Hungarian Natural History Museum in accordance to the regulations on treatment of archaeological human remains in Hungary [35]. No ancient DNA work amplifying human genes was ever done on the premises. Sampling was conducted using measures to prevent contemporary contamination of the specimens. The samples were taken using a no-touch technique with disposable scalpels, from inner organs. These anatomical regions were not previously exposed to the outside environment and therefore were protected from contact with excavators or others that have handled the mummies. The samples were placed in sterile DNA free tubes and stored in room temperature.

**Table 1:** List of samples obtained from the Vác mummies.

Mummy No./ Inventory No.^a^	Name	Year of Death	Age at death	Samples description	Mitochondrial DNA preservation^b^
11/2009.19.11.	Beer Annamária	1807	95	Colon	-
15/2009.19.15.	Fabó Dorottya	1798	66	Liver; colon	+
18/2009.19.18.	Unknown	1831	NA	Back area	-
21/2009.19.21.	Baranyai Alajos	1806	11	Liver	-
28/2009.19.28.	Shöner Anna	1793	55	Chest	-
44/2009.19.44.	Simon Antal	1808	33	Liver; colon	+
51/2009.19.51.	Reihm Vencel	1805	38	Lung; Colon; Liver	+
54/2009.19.54.	Nigrovits Anatal	1803	22	Liver; thorax	-
63/2009.19.63.	Stéger Joachim	1794	37	Bottom; abdomen; liver area	+
65/2009.19.65.	Sándor Terézia	1783	40	Colon; abdomen	+
76/2009.19.76.	Schwartz Mária Terézia	1784	10	Lung; abdomen	-
88/2009.19.88.	Unknown	1770	50–60	Colon; liver	+
96/2009.19.96.	Unknown	1798	20	Testis; liver	-
97/2009.19.97.	Tauber Antónia	1786	37	lungs; colon	+
107/2009.19.107.	Unknown	NA	50–89	right bottom pelvis; colon; liver	+
110/2009.19.110.	Skripetz Klára	1788	18	stomach	-
116/2009.19.116.	Borsodi Terézia	1794	26	Colon; liver	+
124/2009.19.124.	Hummer Anna Mária	1774	50	Stomach	-
145/2009.19.145.	Praun István	NA	30–34	liver	-
254/2009.19.254.	Vaizer Erzsébet	1755	26	left lung; right lung; liver; colon	+

^a^ Numbering and mummy name, age and year of death were kindly supplied by the Hungarian Natural History Museum.

^b^ + positive for mitochondrial D-loop amplification–negative for mitochondrial D-loop amplification.

DNA was extracted in a designated ancient DNA (aDNA) laboratory. To prevent contamination by contemporary DNA the tubes were opened only in a designated UV eradiated hood where DNA extraction was carried out. The aDNA laboratory was physically isolated from the laboratory where modern DNA was used. The procedure was carried out in sterile UV chambers each equipped with separate set of pipettes, disposable sterile tubes, filter tips, molecular biology grade reagents and solutions. Disposable protective clothing was used and changed frequently. Separate UV-irradiated hoods were used for DNA preparation, DNA extractions and PCR preparation. To further minimize contemporary DNA contamination all reagents, tubes and instruments such as disposable scalpel blades were irradiated with UV prior to use. Multiple negative controls for extraction and amplification were included to ensure the authenticity of the aDNA findings. aDNA protocols followed the standard requirements set for the field [36].

### DNA extraction

DNA was extracted from mummified tissue using a modification of guanidine thiocyanate (GuSCN) method developed by Boom R et al. [37] and the silica-based purification method developed by Höss M & Pääbo S [38]. Around 500 mg of tissue was cut into small fragments of approximately 5 mm, placed in a sterile tube containing UV irradiated double distilled water (ddH2O) and incubated at 56°C overnight. The ddH2O were removed and 500 μL of extraction buffer, consisting of 4 M Guanidinium thiocyanate (GuSCN) (Sigma), 0.1 M Tris-HCl pH 6.4 (Sigma), 0.02 M EDTA pH 8 (Biological Industries) and 1.3% Triton X-100 (Sigma), together with 10 μL of 25 mg/ml Proteinase K were added to the tissue. The tissue was further incubated at 56°C for 48 h. The samples were boiled at 94°C for 10 mins and then centrifuged at 13,000 rpm for 3 mins. The supernatant (harboring the extracted DNA) was transferred to a new sterile tube. To extract the DNA from the supernatant 1 mL Sodium iodide (NaI) (6M, Merck), 10 μL linear acryl amide (5mg/ml, Ambion) and 8 μL silica (1g/ml, Sigma) were added. The samples were incubated at 4°C for 1h to enable the binding of the DNA to the silica beads. The silica beads were pelleted by centrifugation and the pellet was washed twice. The first wash was performed using washing buffer containing 0.01 M Tris-HCl pH 7.5, 0.05 M sodium chloride (NaCl) (Frutarum), 0.1 M EDTA pH 8 and 250 μL absolute ethanol (Biolabs) and ddH2O up to a volume of 500 μL. The second wash was with absolute ethanol. The obtained silica beads pellet was air-dried and the aDNA was eluted at 56°C with Tris-EDTA buffer (TE, 1M Tris pH 8 and 0.5 M EDTA pH 8). The extract aDNA was stored at -20°C.

### DNA amplification

Amplification of the APC gene was conducted in a 25μL reaction mixture including 7μL of the aDNA extract with: 10X buffer, 25 mM MgCl_2_, 2.5mM dNTP’s, 10mM BSA (Biolabs), 12 pmol of each primer set and 1.25 units AmpliTaq Gold® 360 DNA polymerase (Applied Biosystems). The aDNA was amplified using a thermocycler with an initial hot-start phase at 95°C for 10 minutes followed by 45 cycles of 15 seconds at 95°C denaturation, 45 seconds annealing at 60–48°C (touch-down) and 45–60 seconds elongation at 72°C. A final extension step at 72°C for 10 minutes was performed following the 45 cycles.

The aDNA extracts were amplified using two primer sets of the APC gene that were designed by the authors of this study using Primer 3.0 software and using two published primer sets of the hyper variable region in the human mitochondrial control region (d-loop) [39]. The APC primer sets were designed to amplify known mutational hot spots on the MCR region. The amplification of the mitochondrial d-loop was used as control to screen-out extracts that might be contaminated with modern DNA of researchers and as a further indication of the aDNA authenticity. The primer sets that were used for amplifications are described in Table 2.

**Table 2:** Primers in this study.

Position in reference	Size of amplicon (bp)	Sequence (5’-3’)	Name	Reference
Mitochondrial (NC_012920.1) 16004–16275	271	Forward: AGCACCCAAAGCTAAGATTC Reverse: CTTTGGAGTTGCAGTTGATG	Mt1 (L16004)	Faerman et al. 2000 [39]
Mitochondrial (NC_012920.1) 16210–16442	232	Forward: CCCATGCTTACAAGCAAGTA Reverse: ATTGATTTCACGGAGGATGG	Mt2 (L16210)	Faerman et al. 2000 [39]
APC (NM_000038.5) 3956–4068	112	Forward: CAGACGACACAGGAAGCAGA Reverse: GTGACACTGCTGGAACTTCG	APC1309	Designed in this study
APC (NM_000038.5) 4377–4484	107	Forward: TGCCACCAAGCAGAAGTAAA Reverse: TCCACTCTCTCTCTTTTCAGCA	APC1450	Designed in this study

### Analysis of obtained sequences

Positive amplifications were sequenced at the DNA Sequencing Unit of the Wise Faculty of Life Sciences, Tel Aviv University using the ABI PRISM® 3100 Genetic Analyzer. The sequences obtained were initially verified using the National Center for Biotechnology Information BLAST algorithm [40]. Chromatograms were individually examined to confirm the quality of sequences, using Sequencher 4.9 [41]. Sense and antisense sequences were generated from each primer set as an additional control to rule out sequencing errors. The Sense and antisense sequences were assembled into a contig in Sequencher 4.9. Each individual contig was visually inspected and verified; any ambiguities were visually resolved. Sequences with poor-quality chromatograms were excluded from the study. A final contig of all sequences was generated using a published reference. Partial mitochondrial profiles were determined for the mummies, for all staff working at the aDNA laboratory and for all sample collectors. To control for contamination during or after sampling, partial mitochondrial profiles and partial APC sequences obtained from mummy samples were compared to the reference sequences and to profiles of the laboratory staff (Tables 3 and 4). Sequences obtained from mummy samples were also compared to each other to control for cross-contamination. We note that the ancient partial mitochondrial profiles and the ancient chromosomal sequences might be influenced by postmortem deamination processes [42]. Thus, some observed transitions might be attributed to DNA damage and not to maternally inherited or chromosomal substitutions respectively. Postmortem DNA damage does not influence our analysis aimed to control for contamination by testing whether mummies share the same SNP pattern as researchers and does not affect any observed transvertions. However, the implications of DNA damage should be considered in case the sequences are used for other purposes.

**Table 3:** Summary of APC sequences obtained from mummies.

Mummy number	Sample type	Sequences obtained within the APC gene	Comparison to Reference sequence (NM_000038.5)
51	Colon	Position 4377–4484 (Primers APC1309)	No differences
63	Liver	Position 3956–4068 (Primers APC1450)	No differences
88	Colon	Position 4377–4484 (Primers APC1309)	APC E1317Q
		Position 3956–4068 (Primers APC1450)	No differences

**Table 4:** Comparison of partial mitochondrial profiles.

Cambridge refseq (NC_012920.1)		Ancient DNA^a^			Lab staff							
Position	Base	Mummy No. 63	Mummy No. 88	Mummy No. 51	M.F	I.H	L.H	R.R.A	E.H.S	G.K.B	H.M	N.Z
16080	A										G	
16124	T	C										
16145	G									A		
16146	A	G										
16176	C									A		
16193	C				T							
16218	C			T								
16219	A				G							
16223	C									T		T
16224	T								C			C
16234	C								T			T
16274	G						A					
16297	T	C										
16298	T	C										
16311	T								C			C
16325	T						C					
16356	T			C							C	
16360	C			T							T	
16362	T				C							
16390	G									A		
Total	20	4	0	3	3	0	2	0	3	4	3	4

^a^ The ancient partial mitochondrial sequences might be influenced by postmortem DNA damage. Thus, C to T and/or G to A transitions might be attributed to postmortem deamination and not to a maternally inherited substitution [42].

## Results

**Fig 1:**
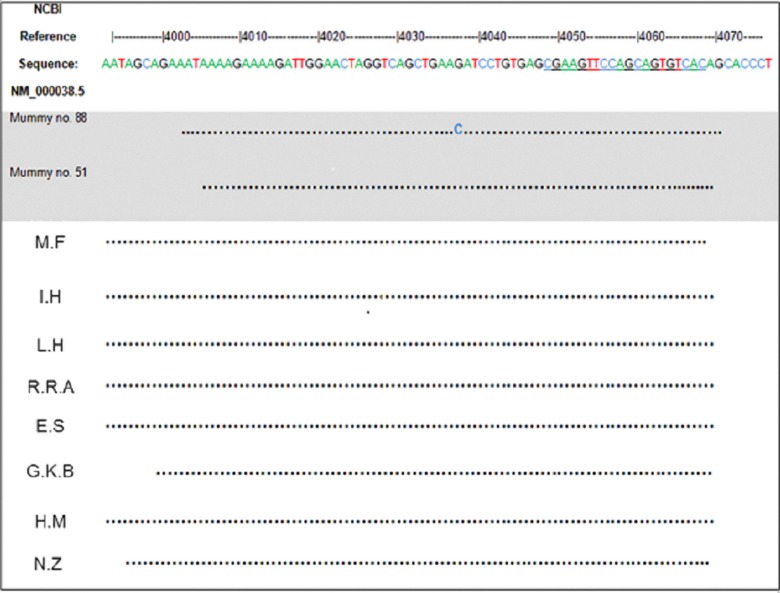
Comparison of partial APC sequences amplified from ancient samples and from the laboratory staff. Partial APC sequences amplified with primers APC1309 compared to the NCBI reference sequence NM_000038.5. Laboratory staff members are indicated with initials. Ancient samples are indicated with a mummy number. The sequencing primer is underlined in the reference sequence. Mummy number 88 is the only carrier of the E1317Q mutation.

**Fig 2:**
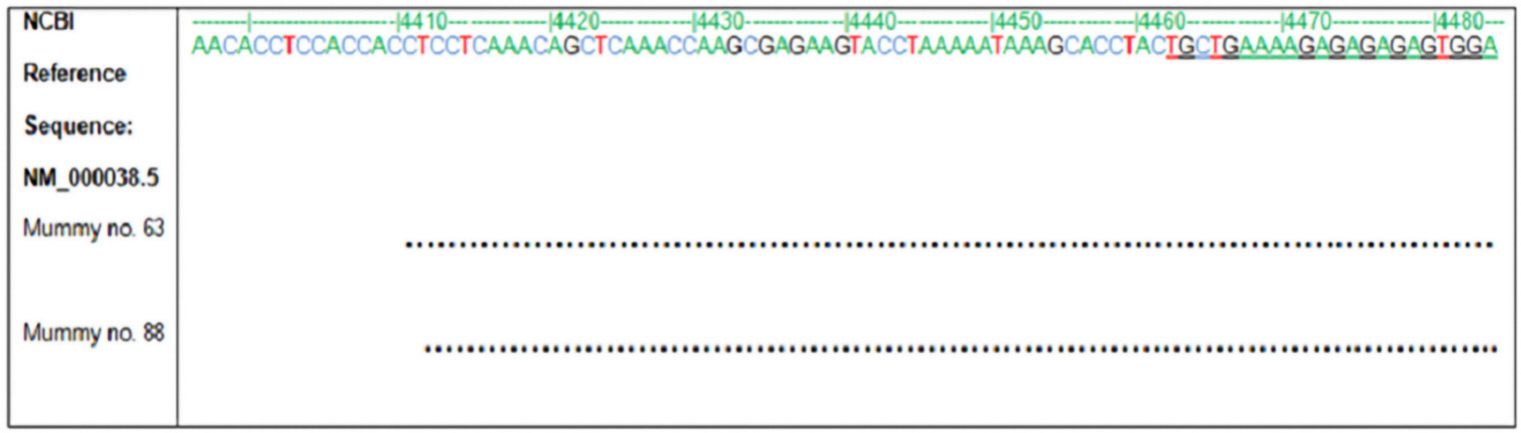
Partial APC sequences amplified from ancient samples. Partial APC sequences that were amplified with primers APC1450 are compared to the NCBI reference sequence NM_000038.5. The sequencing primer is underlined in the reference sequence. These partial APC sequences of the two mummies were identical to the reference sequence that codes for the wild type protein.

**Fig 3:**
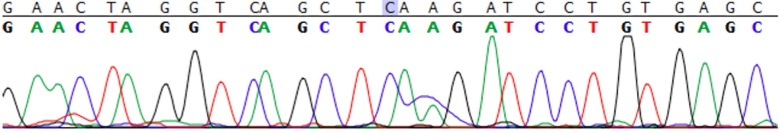
A part of the APC sequence amplified from mummy 88. Highlighted is the homozygous G→C E1317Q missense mutation.

APC is an important tumor suppressor gene. Mutations in APC are strongly associated with the development of colorectal adenomas and carcinomas [6]. To assess the presence of genetic predisposition to colorectal cancer in the pre-industrialization era we attempted to amplify the MCR region of the APC gene from DNA obtained from internal organs of the Vác mummies. Partial sequences of the APC gene MCR region were successfully obtained from three mummies. Two wild type APC sequences were obtained from two mummies numbers 51 and 63 (Table 3, Figs 1 and 2). The sequences of the APC gene MCR (position 4377–4484 and position 3956–4068) acquired from a colon tissue sample of mummy 88 (Table 3, Figs 1 and 2) indicated that this individual was homozygous to a missense mutation in codon 1317 (GAA to CAA) (Figs 1 and 3). This is a known APC genetic variation that substitutes glutamine an uncharged hydrophilic amino acid with glutamate an acidic hydrophilic amino acid (E1317Q). This mutation has been linked with a predisposition to the development of multiple colorectal adenomas and colorectal cancer [43]. The rest of the APC MCR partial sequence for this mummy was identical to the reference (NM_000038.5) that codes for the wild type protein. The sequences of all the researchers that handled the sample or equipment were found to be identical to the reference.

Mitochondrial aDNA was preserved in 50% of the mummies tested (Table 1). Partial mitochondrial profiles (positions 16004–16442) were determined for the 3 mummies for whom the APC MCR sequences were obtained (Table 4). Mummy 51 and 63 showed a unique profile different from the Cambridge reference sequence (NC_012920.1), different from each other and different from the profiles obtained from all handlers. PCR amplification detects mitochondrial DNA with greater sensitivity compared with the detection of chromosomal DNA. This difference in sensitivity is explained mainly by a higher copy number per cell of the mitochondrial DNA [44,45]. Thus, if the sample had been contaminated with modern DNA it is likely modern mitochondrial DNA would have been detected. The unique profiles of the mummies indicate that the sequences obtained for the two mummies are authentic and that there was no cross-contamination between the mummy samples.

The partial mitochondrial sequence of mummy 88 was identical to the Cambridge reference sequence. Among the researchers, only the partial mitochondrial sequences of Dr. Rosin-Arbesfeld (R.R.A) and Prof. Hershkovitz (I.H), who participated in the collection of the samples, were identical to the Cambridge reference sequence as expected due to their European origin. Nevertheless, the observation of the E1317Q mutation could not be due to contamination of mummy 88 by DNA from Dr. Rosin-Arbesfeld or Prof. Hershkovitz since nor they or any other of the researchers, have the APC E1317Q mutation (Fig 1).

## Discussion

We have found the APC nonsense mutation E1317Q in a sample from the large intestine tissue recovered from an 18^th^ century mummy. The wild type APC sequences, at the same position, were obtained from two other mummies from the same collection.

The ability to retrieve genetic materials from ancient tissue was a tremendous step forwards in understanding the evolutionary history of diseases. While most disease aDNA studies focused on the ancient pathogen DNA [46–48], genetic research of cancer in historical populations has been somewhat neglected. There are reports on tumors or benign neoplasms in ancient specimens; some even go back to the dinosaur era [49]. But, these are based mainly on the presence of specific bone lesions or histological studies and not genetic information. In Hungary cases of osteosarcoma; myeloma; and metastatic carcinoma were reported in historical specimens [50–53]. To the best of our knowledge, cancer or mutations associated with cancer have not yet been reported in ancient DNA studies.

The scarcity of reports on tumors in ancient soft tissue remains compared to the large number of autopsies carried out on mummies have led some scholars to hypothesize that malignancies were rare in past populations in comparison with modern times due to the short lifespan of individuals that precluded the development of cancer [15,24]. Conversely, paleopathological reports based on the investigation of skeletal remains suggest tumor rates were similar between the past and modern populations examined [20,23]. Historical accounts indicate that life expectancy was statistically lowered by infant and maternal mortality and yet many individuals did live to a sufficiently advanced age to develop other mid-old age diseases, such as degenerative diseases [15]. Another hypothesis trying to explain the rarity of tumors in ancient soft tissue is that tumors might not be well preserved in mummified tissue postmortem. However, experimental studies show that mummification preserves the features of malignancy [54]. Therefore, in an ancient society lacking surgical intervention, evidence of cancer, if existed in the tissue, should remain in all preserved mummified specimens. The fact archaeological soft tissue specimens are scarce compared to skeletal remains [55] present a challenge for the analysis of ancient cancer related genetic data due to the small sample size. This highlights the importance of accumulation of data from studies such as this, eventually creating a sufficient database for subsequent studies. In recent years, the use of next generation sequencing (NGS) has become common in ancient DNA research [56]. Shotgun sequencing was successfully implemented by Kay et al. 2015 to generate *M*. *tuberculosis* genome sequences from skeletal and soft tissue of the Vác mummies, demonstrating that bacterial whole genome data can be obtained from mummified tissue in general and from the Vác mummy collection in particular [33]. However, based on the data reported by Kay et al. [33], the average fold coverage for the human genomes is very low (not more than 0.09 fold average coverage), indicating targeted DNA enrichment would be required to analyze specific chromosomal regions such as the APC MCR region. Furthermore, human whole genome data has so far not been obtained from mummified tissue. Thus, we chose to employ the classical approach of PCR amplification and direct sequencing to characterize APC gene mutations from the Vác mummies. Since the classic approach is more limited in the ability to address contamination, strict measures were used to prevent DNA contamination during sample processing as described in the methods part; including the comparison of the APC sequences of the mummies with those of all sample handlers. Our findings confirm that the isolation of specific cancer related chromosomal regions from mummified tissue is feasible and might motivate future development of enrichment arrays aimed to capture DNA regions related to malignancy. Such approaches might increase DNA yields for these regions of interest and could be combined with NGS techniques to provide additional means of authentication and a broader outlook on cancer evolution.

Colorectal Cancer arises as the cumulative effect of multiple mutations in many genes allowing the cell to escape from regulatory controls leading to uncontrolled proliferation. These mutations can be inherited or somatic and the latter can be largely affected by environmental factors (e.g. smoking, air pollution and nutrition) [57].

Studies examining the relationship between the APC E1317Q mutation and colorectal cancer have shown different results. While some studies suggest that the mutation contributes to a predisposition to colorectal adenomas and carcinomas with low and variable penetrance [58,59], others claim that the variant is associated with only a moderate increase in risk of colorectal cancer [60–62]. The choice of the control group in some of the studies that did not find a significantly higher risk of colorectal cancer due to E1317Q has been criticized and was proposed to be the cause of the contradiction regarding E1317Q effect on colorectal cancer [43]. Thus, a possible role for E1317Q in colorectal tumor genesis may exist and should be studied further. It has been suggested E1317Q has subtle effects on β-catenin sequestration or degradation but the exact molecular mechanism causing the predisposition for colorectal cancer is unknown [59].

Our data suggest that individual 88 may have had a predisposition for developing colorectal adenomas and carcinomas but we cannot tell whether those conditions actually manifested in this individual. The morphological preservation of the mummified colon tissue was not sufficient to visually differentiate adenomas or carcinomas from normal tissue. The fact that mummy 88 was homozygous for the APC E1317Q sequence somewhat increases the likelihood of manifestation as it is feasible to speculate that homozygousity was caused by a loss of heterozygousity event that is common in neoplasia and is commonly found in colorectal cancer patients that show APC loss of function [63]. As the E1317Q APC variant is rare in the general modern population (0.3%, NCBI SNP database rs1801166) [40], the chances of inheriting one mutated allele from each parent are very low which increases the possibility that a somatic mutation had indeed occurred. Nevertheless, we do not have data on mummy 88's family history or of the allele frequency at 18^th^ century Hungary, therefore we cannot rule out inheritance of homozygousity for E1317Q in this case. Absence of the mutation in tissues taken from other remaining organs, would have confirmed the mutation being somatic and not an inherited germ-line mutation. Unfortunately, the genomic DNA preservation level in the other tissues sampled from mummy 88 (liver) was not sufficient for successful amplification of the genomic APC sequences. In general, somatic mutations in this part of the MCR of the APC gene are more common in modern populations than germ line mutations [64].

Obesity, physical inactivity, a diet high in red or processed meat, alcohol consumption and long-term smoking are specific risk factors for colorectal cancer [3]. These risk factors were less frequent to non-existent in pre-industrialized 18^th^ century Hungary [65,66]. Frequencies of cancer in historical populations, such as the 18^th^ century Hungarian population might be linked with the absence of modern life environmental factors such as tobacco use or pollution [57,67]. Although the APC MCR is a genomic region frequently mutated in modern day population [68], the only mutation detected in the APC MCR sequences obtained from the 3 mummies was E1317Q in mummy 88. This data combined with future data from similar studies spanning different times and locations may elucidate the link between occurrence of colorectal cancer predisposing mutations and historical lifestyle. Human society has undergone enormous lifestyle and environmental changes during the last centuries. The ability to compare the spectrum of historical mutations to the modern spectrum seems important for the understanding of the etiology and molecular pathogenesis of neoplasia. Our data, indicating the presence of a cancer predisposing mutation and possibly cancer in a person from the 18^th^ century combined with data that will be accumulated from future aDNA studies may provide a fuller picture of cancer epidemiology.

## References

[1] Ries L, Wingo P, Miller D, Howe H, Weir H, Rosenberg H, et al The annual report to the nation on the status of cancer, 1973–1997, with a special section on colorectal cancer. Cancer. 2000; 88(10): 2398–2424. 10820364 10.1002/(sici)1097-0142(20000515)88:10<2398::aid-cncr26>3.0.co;2-i

[2] Ferlay J, Autier P, Boniol M, Heanue M, Colombet M, Boyle P. Estimates of the cancer incidence and mortality in Europe in 2006. Annals of Oncology. 2007;18(3): 581–592. 17287242 10.1093/annonc/mdl498

[3] Tarver T. Cancer Facts & Figures 2012. American Cancer Society (ACS) Atlanta. 2012. Journal of Consumer Health on the Internet. 2012; 16(3), 366–367.

[4] Eide TJ. Prevalence and morphological features of adenomas of the large intestine in individuals with and without colorectal carcinoma. Histopathology. 1986; 10(2): 111–118. 3957251 10.1111/j.1365-2559.1986.tb02467.x

[5] Cannon-Albright L, Skolnick MH, Bishop DT, Lee RG, Burt RW. Common Inheritance of Susceptibility to Colonic Adenomatous Polyps and Associated Colorectal Cancers. N Engl J Med. 1988; 319(9): 533–537. 2841598 10.1056/NEJM198809013190902

[6] Joslyn G, Carlson M, Thliveris A, Albertsen H, Gelbert L, Samowitz W, et al Identification of deletion mutations and three new genes at the familial polyposis locus. Cell. 1991; 66(3): 601–613. 1678319 10.1016/0092-8674(81)90022-2

[7] Arminski T, McLean D. Incidence and distribution of adenomatous polyps of the colon and rectum based on 1,000 autopsy examinations. Diseases of the Colon & Rectum. 1964;7(4):249–261. 14176135 10.1007/BF02630528

[8] Horii A, Nakatsuru S, Miyoshi Y, Ichii S, Nagase H, Ando H, et al Frequent Somatic Mutations of the APC Gene in Human Pancreatic Cancer. Cancer Research. 1992; 52(23):6696–6698. 1423316

[9] Horii A, Nakatsuru S, Miyoshi Y, Ichii S, Nagase H, Kato Y, et al The APC Gene, Responsible for Familial Adenomatous Polyposis, Is Mutated in Human Gastric Cancer. Cancer Research. 1992; 52(11):3231–3233. 1317264

[10] Uzawa K, Yoshida H, Suzuki H, Tanzawa H, Shimazaki J, Seino S, et al Abnormalities of the adenomatous polyposis coli gene in human oral squamous-cell carcinoma. International Journal of Cancer. 1994;58(6):814–817. 7927873 10.1002/ijc.2910580611

[11] Nagase H, Nakamura Y. Mutations of the APC (adenomatous polyposis coli) gene. Hum Mutat. 1993;2(6):425–434. 8111410 10.1002/humu.1380020602

[12] Chen T, Hsieh L, Ng K, Jeng L, Chen M. Absence of APC gene mutation in the mutation cluster region in hepatocellular carcinoma. Cancer Lett. 1998;134(1):23–28. 10381126 10.1016/s0304-3835(98)00238-9

[13] Ebbell B. The Ebers Papyrus, the greatest Egyptian medical document Kopenhagen: Leon und Muniksgaard; 1937.

[14] Pain S. The pharaohs' pharmacists. New Sci. 2007;(2634):40–43.

[15] David AR, Zimmerman MR. Cancer: an old disease, a new disease or something in between? Nature Reviews Cancer. 2010;10(10):728–733. 10.1038/nrc2914 20814420

[16] Brothwell D and Sandison AT. Disease in antiquity. A survey of the diseases, injuries and surgery of early populations Springfield: CC Thomas; 1967.

[17] Smith GE, Dawson WR. Egyptian Mummies. UK: G. Allen & Unwin; 1924.

[18] Strouhal E. Tumors in the remains of Ancient Egyptians. Am J Phys Anthropol. 1976;45(3):613–620. 793419 10.1002/ajpa.1330450328

[19] Halperin E. Paleo-Oncology: The Role of Ancient Remains in the Study of Cancer. Perspectives in Biology and Medicine. 2004;47(1):1–14. 15061165 10.1353/pbm.2004.0010

[20] Nerlich AG, Rohrbach H, Bachmeier B, Zink A. Malignant tumors in two ancient populations: an approach to historical tumor epidemiology. Oncol Rep. 2006;16(1):197–202. 16786146

[21] Fornaciari G. Renaissance mummies in Italy. Med Secoli. 1999;11(1):85–105. 11624203

[22] Binder M, Roberts C, Spencer N, Antoine D, Cartwright C. On the Antiquity of Cancer: Evidence for Metastatic Carcinoma in a Young Man from Ancient Nubia (c. 1200BC). PloS one 2014;9(3):e90924 10.1371/journal.pone.0090924 24637948 PMC3956457

[23] Zink A, Rohrbach H, Szeimies U, Hagedorn HG, Haas CJ, Weyss C, et al Malignant tumors in an ancient Egyptian population. Anticancer Res. 1999 Sep-Oct;19(5B):4273–4277. 10628386

[24] Fornaciari G, Giuffra V. Soft tissue tumors in palaeopathology: a review. Pathobiology 2012;79(5):257–267. 10.1159/000337292 22722565

[25] Charlier P, Huynh-Charlier I, Brun L, Devisme L, Catalano P. A 1800-year-old mediastinal mature teratoma. Ann Pathol 2009 2;29(1):67–69. 10.1016/j.annpat.2008.09.051 19233101

[26] Lynnerup N. Mummies. Am J Phys Anthropol 2007;134(S45):162–190. 18046750 10.1002/ajpa.20728

[27] Szikossy I, Kustár Á, Guba Z, Kristóf L, Pap I. Naturally mummified corpses from the Dominican Church in Vác, Hungary In: Wieczorek A R, editor. Mummies of the World. American Exhibition, Reiss-Engelhorn-Museum, Mannheim/Prestel/Munich/Berlin/London/New York; 2010 p. 160–171.

[28] Pap I, Susa E, Jozsa L. Mummies from the 18th-19th century Dominican church of Vac Hungary. Acta Biology Szeged. 1997(42):107–112.

[29] Chan JZ, Sergeant MJ, Lee OY, Minnikin DE, Besra GS, Pap I, et al Metagenomic Analysis of Tuberculosis in a Mummy. N Engl J Med. 2013; 369(3):289–290. 10.1056/NEJMc1302295 23863071

[30] Fletcher HA, Donoghue HD, Holton J, Pap I, Spigelman M. Widespread occurrence of Mycobacterium tuberculosis DNA from 18^th^-19^th^ century Hungarians. Am J Phys Anthropol. 2003;120(2):144–152. 12541332 10.1002/ajpa.10114

[31] Spigelman M, Donoghue HD, Abdeen Z, Ereqat S, Sarie I, Greenblatt CL, et al Evolutionary changes in the genome of Mycobacterium tuberculosis and the human genome from 9000 years BP until modern times. Tuberculosis 2015. 10.1016/j.tube.2015.02.022 25771203

[32] Pap I, Józsa L, Repa I, Bajzik G, Lakhani S, Donoghue H, et al 18–19th century tuberculosis in naturally mummified individuals (Vác, Hungary) In: Pálfi G, Dutour O, Deák J, Hutás I, editors. Tuberculosis: Past and Present Budapest: Szeged; 1999 p. 419–428.

[33] Kay GL, Sergeant MJ, Zhou Z, Chan JZ, Millard A, Quick J, et al Eighteenth-century genomes show that mixed infections were common at time of peak tuberculosis in Europe. Nature communications 2015;6. 10.1038/ncomms7717 PMC4396363 25848958

[34] Fletcher HA, Donoghue HD, Taylor GM, van der Zanden AG, Spigelman M. Molecular analysis of Mycobacterium tuberculosis DNA from a family of 18th century Hungarians. Microbiology 2003 1;149(Pt 1):143–151. 12576588 10.1099/mic.0.25961-0

[35] Pap I, Pálfi G. Hungary/Magyar Köztársaság In: Márquez-Grant N, Fibiger L, editors. The Routledge Handbook of Archaeological Human Remains and Legislation: An international guide to laws and practice in the excavation and treatment of archaeological human remains. London; New York: Routledge; 2011 p. 185–201.

[36] Cooper A, Poinar HN. Ancient DNA: Do It Right or Not at All. Science. 2000; 289(5482):1139–1139. 10970224 10.1126/science.289.5482.1139b

[37] Boom R, Sol C, Salimans M, Jansen C, Wertheim-van Dillen P, Van der Noordaa J. Rapid and simple method for purification of nucleic acids. J Clin Microbiol. 1990; 28(3):495–503. 1691208 10.1128/jcm.28.3.495-503.1990 PMC269651

[38] Höss M, Pääbo S. DNA extraction from Pleistocene bones by a silica-based purification method. Nucleic Acids Research 1993 8 11;21(16):3913–3914. 8396242 10.1093/nar/21.16.3913 PMC309938

[39] Faerman M, Nebel A, Filon D, Thomas MG, Bradman N, Ragsdale BD, et al From a dry bone to a genetic portrait: A case study of sickle cell anemia. Am J Phys Anthropol. 2000;111(2):153–163. 10640943 10.1002/(SICI)1096-8644(200002)111:2<153::AID-AJPA2>3.0.CO;2-O

[40] Altschul S, Gish W, Miller W, Myers E, Lipman DJ. Basic local alignment search tool. J Mol Biol. 1990; 215(3):403–410. 2231712 10.1016/S0022-2836(05)80360-2

[41] Sequencher® version 5.3 sequence analysis software, Gene Codes Corporation, Ann Arbor, MI USA. http://www.genecodes.com

[42] Briggs AW, Stenzel U, Johnson PLF, Green RE, Kelso J, Prüfer K, et al Patterns of damage in genomic DNA sequences from a Neandertal. Proceedings of the National Academy of Sciences 2007 9 11;104(37):14616–14621. 10.1073/pnas.0704665104 PMC1976210 17715061

[43] Hahnloser D, Petersen GM, Rabe K, Snow K, Lindor NM, Boardman L, et al The APC E1317Q Variant in Adenomatous Polyps and Colorectal Cancers. Cancer Epidemiology Biomarkers & Prevention. 2003; 12(10):1023–1028. 14578138

[44] Schwarz C, Debruyne R, Kuch M, McNally E, Schwarcz H, Aubrey AD, et al New insights from old bones: DNA preservation and degradation in permafrost preserved mammoth remains. Nucleic Acids Research 2009 6 01;37(10):3215–3229. 10.1093/nar/gkp159 19321502 PMC2691819

[45] Alonso A, Martin P, Albarran C, Garcia P, Garcia O, de Simon LF, et al Real-time PCR designs to estimate nuclear and mitochondrial DNA copy number in forensic and ancient DNA studies. Forensic Sci Int 2004 1/28;139(2–3):141–149. 15040907 10.1016/j.forsciint.2003.10.008

[46] Hershkovitz I, Donoghue H, Minnikin D, Besra G, Lee O. Detection and Molecular Characterization of 9000-Year-Old Mycobacterium tuberculosis from a Neolithic Settlement in the Eastern Mediterranean. PLoS ONE. 2008; 3(10): 3426 10.1371/journal.pone.0003426 PMC2565837 18923677

[47] Schuenemann VJ, Singh P, Mendum TA, Krause-Kyora B, Jäger G, Bos KI, et al Genome-Wide Comparison of Medieval and Modern Mycobacterium leprae. Science 2013; 341(6142):179–183. 10.1126/science.1238286 23765279

[48] Kahila Bar-Gal G, Kim MJ, Klein A, Shin DH, Oh CS, Kim JW, et al Tracing hepatitis B virus to the 16th century in a Korean mummy. Hepatology. 2012;56(5):1671–1680. 10.1002/hep.25852 22610996

[49] Hershkovitz I, Rothschild BM. Neoplastic conditions (Cancer) in Dinosaurs. McGraw-Hill Yearbook of Science and Technology 2001:294.

[50] Marcsik A, Szathmáry L, Finnegan M. Multiple myeloma and metastatic skeletal lesions in osteoarchaeology samples. Journal of Paleopathology 2002;14(2):77–86.

[51] Jozsa L, Fóthi E. Juxtacortical osteosarcoma on tibia and fibula from a medieval cemetery of Budapest. Journal of Paleopathology 2003;15(1):23–32.

[52] Molnár E, Marcsik A, Bereczki Z, Schmidt-Schultz TH, Schultz M, Pálfi G. Malignant tumors in osteoarchaeological samples from Hungary. Acta biologica Szegediensis 2009;53:117–124.

[53] Pálfi G. The occurrence of bone tumors in the anthropological remains belonging to the Székkutas-Kápolnadűlő cemetery (Hungary) of the Late Avar period. Acta Biol Szeged 1989;35:207–220.

[54] Zimmerman MR. An experimental study of mummification pertinent to the antiquity of cancer. Cancer. 1977; 40(3): 1358–1362. 902245 10.1002/1097-0142(197709)40:3<1358::aid-cncr2820400354>3.0.co;2-j

[55] Marota I, Rollo F. Molecular paleontology. Cellular and Molecular Life Sciences. 2002;59(1):97–111. 11846037 10.1007/s00018-002-8408-8 PMC11337491

[56] Marciniak S, Klunk J, Devault A, Enk J, Poinar HN. Ancient human genomics: the methodology behind reconstructing evolutionary pathways. J Hum Evol 2015;79:21–34. 10.1016/j.jhevol.2014.11.003 25601038

[57] Lilienfeld AM, Pedersen E, Dowd JE editors. Cancer epidemiology: methods of study xiii ed. Baltimore: Johns Hopkins press; 1968.

[58] Lamlum H, Al Tassan N, Jaeger E, Frayling I, Sieber O, Reza FB, et al Germline APC variants in patients with multiple colorectal adenomas, with evidence for the particular importance of E1317Q. Human Molecular Genetics. 2000; 9(15):2215–2221. 11001924 10.1093/oxfordjournals.hmg.a018912

[59] Frayling IM, Beck NE, Ilyas M, Dove-Edwin I, Goodman P, Pack K, et al The APC variants I1307K and E1317Q are associated with colorectal tumors, but not always with a family history. Proceedings of the National Academy of Sciences. 1998; 95(18):10722–10727. 10.1073/pnas.95.18.10722 PMC27962 9724771

[60] Polakis P. The adenomatous polyposis coli (APC) tumor suppressor. Biochimica et Biophysica Acta (BBA)—Reviews on Cancer. 1997; 1332(3): 127–147. 10.1016/s0304-419x(97)00008-5 9196022

[61] Popat S, Stone J, Coleman G, Marshall G, Peto J, Frayling I, et al Prevalence of the APC E1317Q variant in colorectal cancer patients. Cancer Lett. 2000;149:203–206. 10737725 10.1016/s0304-3835(99)00360-2

[62] Figer A, Irmin L, Geva R, Flex D, Sulkes A, Friedman E. Genetic analysis of the APC gene regions involved in attenuated APC phenotype in Israeli patients with early onset and familial colorectal cancer. Cancer. 2001;85:523. 10.1054/bjoc.2001.1959 PMC2364108 11506490

[63] Takahashi T, Nau M, Chiba I, Birrer M, Rosenberg R, Vinocour M, et al p53: a frequent target for genetic abnormalities in lung cancer. Science. 1989; 246(4929):491–494. 2554494 10.1126/science.2554494

[64] M. Sieber O, P. Tomlinson I, Lamlum H. The adenomatous polyposis coli (APC) tumour suppressor–genetics, function and disease. Mol Med Today. 2000; 6(12):462–469. 11099951 10.1016/s1357-4310(00)01828-1

[65] Őri P. Patterns of demographic behaviour in late 18^th^ century Hungary. Demográfia. 2005; 48: 43–76.

[66] Braudel F. Civilization and capitalism 15th-18th century 1st ed. USA: University of California press ed.; 1992.

[67] Roberts C, Manchester K. The Archaeology of Disease. 3rd ed. Sparkford, UK: Sutton; 2005.

[68] Nishisho I, Nakamura Y, Miyoshi Y, Miki Y, Ando H, Horii A, et al Mutations of chromosome 5q21 genes in FAP and colorectal cancer patients. Science. 1991; 253(5020):665–669. 1651563 10.1126/science.1651563

